# Cytotoxic, genotoxic, and toxicogenomic effects of heated tobacco products and cigarette smoke in human primary keratinocytes

**DOI:** 10.18332/tid/152510

**Published:** 2022-09-30

**Authors:** Yoshihisa Morishita, Shogo Hasegawa, Shin Koie, Sei Ueda, Satoru Miyabe, Satoshi Watanabe, Mitsuo Goto, Hitoshi Miyachi, Shuji Nomoto, Toru Nagao

**Affiliations:** 1 Department of Maxillofacial Surgery, School of Dentistry, Aichi Gakuin University Graduate School of Medicine, Nagoya, Japan; 2 Department of Surgery, School of Dentistry, Aichi Gakuin University Graduate School of Medicine, Nagoya, Japan

**Keywords:** tobacco, smoking, carcinogenesis, Heated Tobacco Products

## Abstract

**INTRODUCTION:**

Heated tobacco products (HTPs) appear to be less harmful to health than conventional cigarettes (CCs). However, limited analytical data are available to support this claim. This study aimed to compare the cytotoxic, genotoxic, and toxicogenomic effects of HTPs and CCs in carcinogenesis via multistep gene mutations in the oral mucosal cells.

**METHODS:**

Cigarette smoke extract (CSE) was obtained from HTPs and CCs. Primary human oral keratinocytes (HOKs) were treated with 5% and 20% CSE from HTPs and CCs. Cell survival rate assays were performed after 6, 12, and 24 h. After 6 h, DNA double-strand breaks (DSBs) were evaluated using anti-γH2AX antibodies with immunohistochemistry. mRNAs expressions of mediator of DNA damage checkpoint 1 (MDC1) and ataxia telangiectasia and Rad3-related protein (ATR), were analyzed. Expressions of miR-22 and miR-185 were analyzed because miR-22 targets MDC1 and miR-185, ATR.

**RESULTS:**

The HOKs had equivalent survival rates after exposure to the same concentrations of CSE from CCs and HTPs. HTPs increased foci formation of γH2AX in HOKs, as did CCs (without CSE vs 20% HTP, p<0.05; without CSE vs 20% CC, p<0.05). Expressions of MDC1 and ATR decreased in cells exposed to CSE from CCs and HTPs (MDC1: without CSE vs 20% HTP, p<0.05; without CSE vs 20% CC, p<0.05; ATR: without CSE vs 20% HTP, p<0.05; without CSE vs 20% CC, p<0.05). Expressions of miR-22 and miR-185 were not significantly increased when exposed to CSE from CCs or HTPs.

**CONCLUSIONS:**

HTPs and CCs had similar cytotoxic effects. HTPs are genotoxic, can cause DSBs, and have toxicogenomic damage because they inhibit the MDC1 and ATR-CHK1 DNA repair pathways in the oral mucosa. The miRNA-mRNA axis was not related to these inhibitions.

## INTRODUCTION

The World Health Organization’s International Agency for Research on Cancer concluded that cigarette smoking is the leading cause of death from oral and oropharyngeal cancers^1,2^. Cigarette smoke contains thousands of chemicals and compounds, including oxidants and free radicals, which induce DNA damage^3^. Accumulated DNA damage can lead to mutations and chromosomal rearrangements, resulting in genomic instability, which is associated with carcinogenesis. Among the types of DNA damage, double-strand breaks (DSBs) are the most severe and difficult to repair. Several studies have linked smoking with DSB formation^4-6^.

Mediators of DNA damage checkpoint 1 (MDC1) and ataxia telangiectasia, and Rad3-related (ATR) checkpoint kinase 1 (CHK1) are important components of the DNA damage response (DDR) mechanism and have been reported to induce the assembly of DDR proteins at DNA damage sites^7-9^. DSBs activate DDR by triggering the kinase activity of the ataxia-telangiectasia mutated (ATM), thereby initiating a signaling cascade wherein the histone H2AX, located at DSB sites, is phosphorylated (γH2AX), and other DDR factors, including the adaptor protein MDC1, are recruited. MDC1 amplifies ATM signaling activity, leading to a higher percentage of γH2AX proteins and contributing to the recruitment and retention of additional DDR factors at DNA damage sites^10,11^. Several related microRNAs (miRNAs) have been associated with MDC1 and ATR, and the regulation of the modulation of MDC1 in ATM and ATR-CHK1 cell signaling pathways has also been identified.

A new brand of heated tobacco products (HTPs), which have been shown to reduce exposure to harmful substances that are only produced at combustion temperatures, has been reported^12^. HTPs can generate aerosols by heating tobacco leaf sheets without burning them. Although the relationship between smoking and carcinogenicity has been widely accepted, the carcinogenicity of HTPs and their relationship with DNA repair genes remain to be elucidated. Compared with conventional cigarettes (CCs), HTPs reduce the emission levels of nine specific toxicants [e.g. CO; 1,3-butadiene; benzene; benzo[a] pyrene; N-nitrosonornicotine (NNN); and 4-(methylnitrosamino)-1-(3-pyridyl)-1-butanone (NNK)] from cigarettes, according to the mandates of the World Health Organization (WHO) Study Group on Tobacco Product Regulation^13,14^.

In recent years, there have been diverse reports on the association between HTPs and respiratory diseases, such as lung cancer, genotoxicity in rat’s lung, as well as adverse cardiovascular effects and hepatotoxicity in rats^15-17^; however, reports on oral cavity cancer are lacking. There is evidence of a relationship between oral cancer and smoking. The oral mucosa is usually the first part of a consumer’s body to be exposed to the components of tobacco products, making it a frequent site for cytotoxicity, genotoxicity, and toxicogenomic and clinical effects of tobacco use^18^. Because a rapid increase in HTP use has been noted in young people, it is important to understand the risk of oral mucosal carcinogenesis; hence, research on HTP carcinogenesis is urgently needed to educate the public.

In this study, we aimed to: 1) obtain novel evidence on the cytotoxicity, genotoxicity, and toxicogenomic effects of HTPs versus CCs to determine whether HTPs are involved in the development of oral cavity cancer; and 2) investigate the relationship between HTPs and cytotoxicity by evaluating cell proliferation and between HTPs and genotoxicity by focusing on DSBs. We also performed a comprehensive gene expression analysis to investigate toxicological effects, focusing on the miRNA-messenger RNA (mRNA) axis.

## METHODS

### Preparation of cigarette smoke extract solutions

Solutions of cigarette smoke extract (CSE) were prepared in a vacuum vessel containing 100 mL of phosphate-buffered saline (PBS) that was prewarmed to 37°C. The mainstream smoke was drawn through PBS using a vacuum^19,20^. CSEs were obtained from CCs and HTPs by burning and heating them, respectively, using a commercially available device. The CCs were consumed fully, while 14 puffs were taken from the HTPs in compliance with the manufacturer’s recommendation. This device cannot contain more than 14 puffs because of product limitations. Two types of CSE were obtained by vacuuming CCs and HTPs separately. CC solution was obtained by vacuuming 100 mL of PBS solution until CCs were fully consumed. In contrast, HTP solution was obtained by vacuuming 100 mL of PBS solution until 14 puffs of HTPs were consumed. To check and maintain consistency in the different lots of CSE, gas chromatography–mass spectrometry analysis was requested from Japan Food Research Laboratories. CCs and HTPs had similar nicotine concentrations. We diluted the CSEs of CC and HTP with an oral keratinocyte medium (ScienCell Research Laboratories, Carlsbad, CA, USA) to obtain 5% and 20% concentrations, which were calculated using the following equation: (mL CSE solution ÷ total mL) × 100. The total mL in this equation is the sum of the volumes of the CSE solution (mL) and oral keratinocyte medium (mL). The component analysis results showed that CSE solutions of 5% and 20% approximately corresponded to exposures associated with smoking 0.4 packs and 1 pack of cigarettes per day, respectively.

### Cell culture

Primary human oral keratinocytes (HOKs) were purchased from ScienCell Research Laboratories (Carlsbad, CA, USA) and cultured in an oral keratinocyte medium containing 5 mL oral keratinocyte growth supplement and 5 mL penicillin/streptomycin solution. All cells were cultured in an incubator at 37°C, with an atmosphere of 5% CO_2_/95% air. HOKs were grown to 80% confluence in CELLSTAR Advanced TC 100×20 mm tissue culture dishes (Greiner Bio-One International GmbH, Japan).

### Cell proliferation

CSE solutions (5% and 20%) were added to the tissue culture dishes, and control cells were cultured in an HOK medium. Live/dead staining was performed to count the number of live cells after 6, 12, and 24 h (n=3–4 per group). Live/dead staining was performed using a One Cell Counter (Biomedical science, Japan) after adding trypan blue solution (FUJIFILM Wako Chemical Corporation, Japan). The cell survival rate was calculated by dividing the cell density at 6, 12, and 24 h by the cell density of the control. Comparisons were made between the same concentrations of each CSE (5% HTP vs 5% CC; 20% HTP vs 20% CC) at each time point.

### Immunohistochemistry

Immunofluorescence was used to measure DNA damage at the histone level by quantifying the γH2AX foci in HOKs. The cells were stained with an anti-γH2AX antibody (DOJINDO, Japan) according to the manufacturer’s instructions. Cells were seeded on a coated μ-Slide 8 well plate (NIPPON Genetics, Japan), grown to 80% confluence, and exposed to CSE for 6 h. Control cells were cultured in an HOK medium. The cells were washed thoroughly with PBS and 250 mmol/L HEPES (pH 7.4) containing 4% PFA and 0.1% Triton X-100 for fixation. Subsequently, the cells were incubated at room temperature for 5 min. The supernatant was discarded, and the cells were washed twice with PBS. PBS containing 1% Triton X-100 was added, and the cells were incubated at room temperature for 20 min. The cells were incubated with the primary antibody for 1 h at room temperature and subsequently incubated with the secondary antibody containing Green Fluorescent Protein (GFP) for 1 h. Fluorescent images were captured using a fluorescence microscope (BZ-X710; Keyence, Japan). γH2AX foci-positive cells were counted per 1 focus (n=4–5 per group). Sample concentrations were compared with the control (control vs 5% HTP; control vs 20% HTP; control vs 5% CC; control vs 20% CC).

### mRNA microarray

Total RNA was extracted from the cell lines 6 h after adding CSE using the RNeasy Mini Kit (Qiagen, Germany). The control group was cultured in an HOK medium without exposure to CSE. RNA quality was assessed using an Agilent 2100 Bioanalyzer (Agilent Technologies, Santa Clara, CA, USA). Reverse transcription was performed using the miRCURY LNA RT Kit (Qiagen, Germany), and mRNA expression profiling of RNA samples was performed using a Human DNA Damage Signaling RT2 Profiler PCR Array (Qiagen, Germany). This array profiled the expression of 84 genes involved in DNA damage signaling pathways. The genes featured were associated with the ATR-CHK1/ATM signaling network and transcriptional targets of DDR.

### Real-time RT-qPCR

*mRNAs*

To confirm the mRNA microarray data, SYBR Green-based reverse transcription-quantitative polymerase chain reaction (RT-qPCR) assays were performed. Total RNA was extracted using the Rneasy Mini Kit (Qiagen, Germany) for cell lines. MDC1 and ATR expression was measured using the One Step TB Green PrimeScript^TM^ PLUS RT-PCR Kit (Takara, Japan) according to the manufacturer’s instructions. RT-qPCR was performed using the Applied Biosystems^TM^ StepOnePlus^TM^ Real-Time PCR System (Thermo Fisher Scientific, USA). The relative mRNA expression was normalized to that of glyceraldehyde-3-phosphate dehydrogenase (GAPDH). Relative expression was calculated using the comparative threshold (Ct) method (n=3)^21^. The following primers from Bio-Rad Laboratories (CA, USA) were used for RT-qPCR: MDC1 (UniqueAssayID: qHsaCED0037094), ATR (UniqueAssayID: qHsaCID0022638), and GAPDH (UniqueAssayID: qHsaCED0038674). The expression was compared between each sample concentration and control (control vs 5% HTP; control vs 20% HTP; control vs 5% CC; control vs 20% CC).

*miRNAs*

Because miR-22 and miR-185 have been shown to target MDC1 and ATR in previous reports^22,23^, we performed real-time RT-qPCR to confirm the variation in miRs. SYBR Green-based RT-qPCR assay for miRNA was performed. Reverse transcription was performed using the miRCURY LNA RT Kit (Qiagen, Germany). The expression of hsa-miR-22-3p (YP00204606) and hsa-miR-185-5p (YP00206037) were measured using the miRCURY LNA miRNA PCR Starter Kit (Qiagen, Germany) according to the manufacturer’s instructions. RT-qPCR was performed using the Applied Biosystems^TM^ StepOnePlus^TM^ Real-Time PCR System (Thermo Fisher Scientific, USA). The small RNA hsa-miR-103a-3p (YP00204063) was used as an internal control. Relative expression was calculated using the comparative threshold (Ct) method (n=3)^21^.

### Statistical analysis

The Wilcoxon test was used to determine the statistical differences between controls and samples. Data are expressed as median and interquartile range (IQR), and statistical significance was set at p<0.05.

## RESULTS

### Cell survival rate

**Figure 1:**
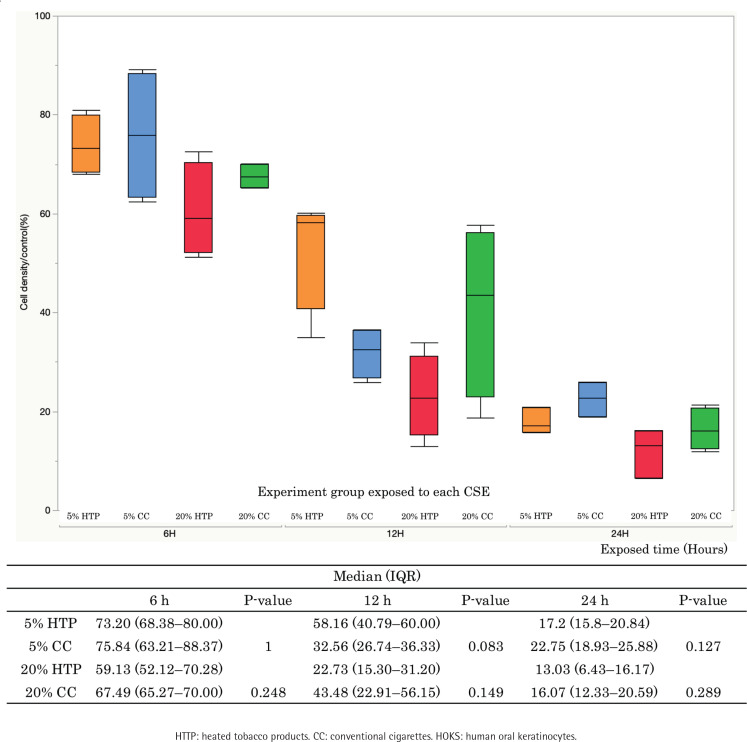
Cell survival rate. The survival rate of HOKs decreased in a concentration-dependent manner in both HOKs exposed to CSE from HTPs and CCs (n=3–4). There were no significant differences between 5% HTP and 5% CC or between 20% HTP and 20% CC at each time point

The survival rate of HOKs decreased in a concentration-dependent manner when HOKs were exposed to CSE from both CCs and HTPs. There were no significant differences in cell survival between the 5% CC and 5% HTP solutions (6 h: p=1.000; 12 h: p=0.083; 24 h: p=0.127), and between the 20% CC and 20% HTP solutions (6 h: p=0.248; 12 h: p=0.149; 24 h: p=0.289) at each time point (Figure 1).

### DSBs

**Figure 2:**
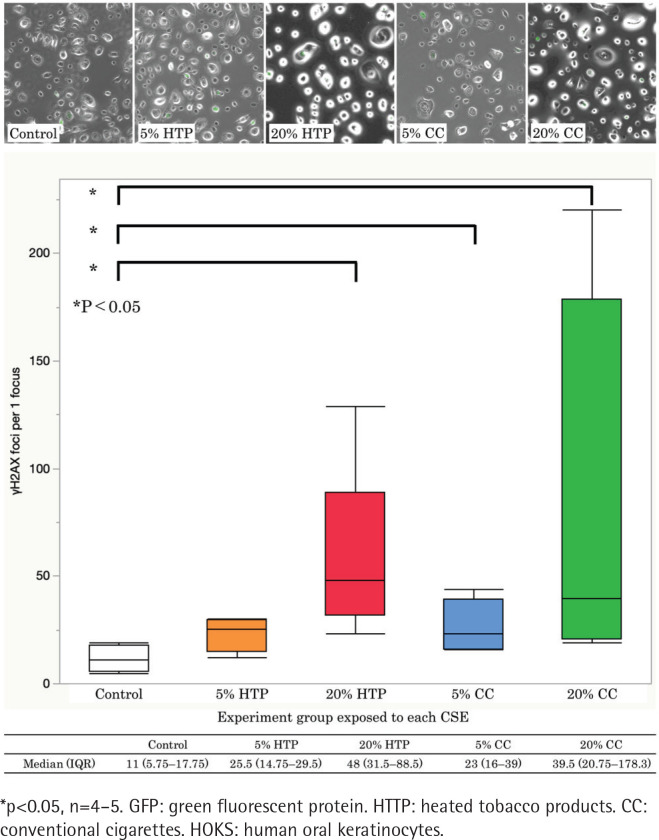
GFP-labeled γH2AX and γH2AX foci-positive cells were counted per 1 focus immunofluorescence staining of γH2AX (green) in HOKs. The cells were subsequently exposed to 5% and 20% CSE solutions for 6 h. γH2AX foci were significantly higher in the HTP and CC groups than in the control group

The formation of γH2AX foci increased in a CSE concentration-dependent manner. The number of γH2AX foci was significantly higher in the CC and HTP groups than in the controls (p<0.05). γH2AX foci in the 20% HTP sample had a median of 48 compared with that in the control and the 20% CC sample, which had medians of 11 and 39.5, respectively (Figures 2 A and B).

### mRNA microarray

MDC1 expression in the 20% CC solution was downregulated by a 0.15-fold change compared with that in the control. Similarly, MDC1 expression was downregulated 0.18-fold in the 20% HTP solution. ATR expression was downregulated by a 0.30-fold change in the 20% CC solution and a 0.36-fold change in the 20% HTP solution compared with that in the control.

### Real-time RT-qPCR

*mRNAs*

**Figure 3:**
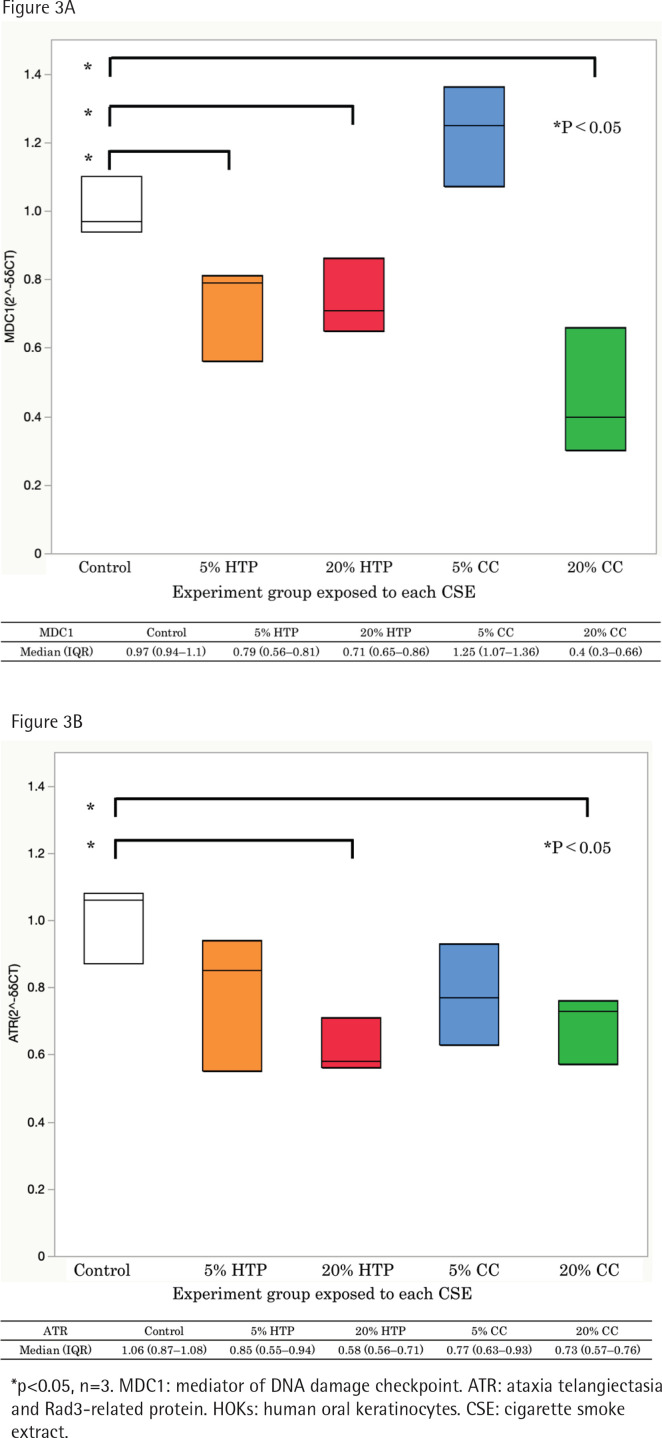
The level of MDC1 (A) and ATR mRNAs (B) in HOKs cells exposed to CSE solutions for 6 h using real-time qPCR

The expressions of MDC1 and ATR mRNAs were significantly decreased in cells exposed to CSE from CCs and HTPs compared with that in the control (p<0.05). The expression of MDC1 mRNA in the 20% HTP sample had a median of 0.71 compared with that in the control and the 20% CC sample, which had medians of 0.97 and 0.40, respectively (Figures 3 A and B).

*miRNAs*

The expressions of miR-22 and miR-185 were not significantly increased in cells exposed to the CSEs of CCs and HTP compared with that noted in the control. The expression of miR-22 in the 20% HTP sample had a median of 8.38 compared with that noted in the control and the 20% CC sample, which had medians of 5 and 8.38, respectively. Furthermore, the expression of miR-185 in the 20% HTP sample had a median of 0.59 compared with that noted in the control and the 20% CC sample, which had medians of 0.99 and 0.16, respectively.

## DISCUSSION

HTPs are marketed as having reduced harmful substances because of the absence of combustion, which is present in CCs^12^. However, it has been reported that HTPs contain the same amount of nicotine and substances as CCs^24^. Furthermore, according to a report comparing the cytotoxicity of aerosols from HTPs and CCs *in vitro*, HTPs were observed to be as toxic as cigarettes in various cells, including respiratory cells^25,26^. In this study, we focused on the oral mucosa because it is the first part of the human body that is exposed to smoking components. We selected HOKs, which are suitable for research related to smoking-induced cytotoxicity and carcinogenesis. Because HOKs are not immortalized, they are similar to normal oral mucosal cells *in vivo*. The cell survival rate assay suggested that HTPs may have the same or higher cytotoxicity than CCs in the oral mucosa.

Highly toxic DSBs are induced by various chemical and physical DNA-damaging agents, such as smoking^4-6^. Unresolved DSBs have been implicated in atherosclerotic cardiovascular disease, neurodegenerative disorders, and cancers^27,28^. γH2AX has been described as a sensitive marker of DSBs^29^. Our results suggest that HTPs cause DSBs in oral mucosal cells, similar to CCs.

Regarding the DNA repair process, we focused on MDC1 and ATR according to the mRNA microarray results and performed real-time RT-qPCR analysis.

MDC1 and ATR levels decreased in HTPs and CCs at 6-h exposure. Genotoxic, toxicogenomic effects were suspected because of the increased DSB, which is related to γHA2X, and the decreased expressions of MDC1 and ATR. The cell survival rate at 12 and 24 h indicate that many cells will die, whereas others could progress toward carcinogenesis. HTPs may inhibit the MDC1 and ATR-CHK1 DNA repair pathways, similar to CCs. MDC1 and ATR play essential roles in suppressing genomic instability and tumorigenicity. It has been suggested that HTPs may be carcinogenic owing to toxicogenomic instability. These findings support the hypothesis that smoking-related mRNAs play a critical role in smoking-related oral cancer.

In recent years, miRNAs, which target mRNAs to regulate gene expression, have attracted considerable attention in the field of carcinogenesis. Here, we focused on miRNAs as mRNA regulators. DNA repair pathways are regulated by various miRNAs^30,31^. miRNAs, which are short, non-coding RNAs 20–22 nucleotides in length, regulate gene expression at the posttranscriptional level by interacting with the 3'-untranslated regions (3'-UTRs) of a target gene^32,33^. They are involved in a wide range of biological functions and can function as oncogenes or tumor suppressors according to the functions of their target genes^34^. According to previous reports, miR-22 targets MDC1 and miR-185, ATR^22,23^. Therefore, we performed RT-qPCR to assess miRNA expression. However, there were no significant differences in the expressions of miR-22 and miR-185, and the results showed no miRNA-mRNA axis. miRNAs bind with incomplete homology and have multiple target mRNAs; therefore, it was included as a parameter in this study to strengthen the investigation. Further, in preliminary experiments using mRNA, miRNA suggested an association; however, we should have increased the number of groups and performed a cluster analysis to identify miRNAs that are expressed by CSE exposure.

Finally, regarding toxicants, compared with CCs, HTPs reduce the emission levels of nine specific toxicants [e.g. CO; 1,3-butadiene; benzene; benzo[a]pyrene; N-nitrosonornicotine (NNN); and 4-(methylnitrosamino)-1-(3-pyridyl)-1-butanone (NNK)] from cigarettes according to the mandates of the WHO Study Group on Tobacco Product Regulation^13,14^. Philip Morris International, Inc. (PMI) reported that the levels of 40 out of 93 harmful and potentially harmful constituents (HPHCs) on the Food and Drug Administration (FDA) HPHC list were lower in HTPs than in CCs.

However, the levels of 56 other constituents, which are not included in the FDA’s list of HPHCs, were higher in HTPs; 22 were >200% higher and seven were >1000% higher in HTPs than in CCs. A number of these substances cause significant cytotoxicity, such as: α,β-unsaturated carbonyl compounds (e.g. 2-cyclopentene-1,4-dione); 1,2-dicarbonyl compounds (e.g. cyclohexane, 1,2-dioxo-); furans [e.g. 2 (5H)-furanone]; and epoxides (e.g. anhydrolinalool oxide)^12,35^. Similarly, genotoxic compounds, including formaldehyde, acetaldehyde, and acrolein, via dehydration and oxidation of the humectants, propylene glycol and glycerin are generated by heating HTPs device^36,37^.

The concentrations of some toxicants are lower in HTPs than in CCs. In contrast, the levels of some cytotoxic and genotoxic substances generated increase when heating the device, and it is assumed that they cause similar effects as CCs. Our research suggests that cytotoxicity, genotoxicity, and toxicogenomic effects of HTPs are not mitigated in the oral mucosa.

### Strengths and limitations

The strengths of the current study include the fact that the concentration of CSE exposed to cells can be easily controlled. In addition, the cells were evenly exposed to CSE; however, to clearly observe the effects of cigarette smoke *in vitro*, the ‘air-liquid interface culture’ method is worth considering. This method, which is applicable to *in vitro* models, involves oculturing cells in contact with the external air of cigarette smoke provided by VITROCELL Exposure Systems (Vitrocell systems, Germany).

At 6-h exposure, the accumulation of DNA damage was suspected because of increased DSB, which is related to γHA2X, and the decreased expressions of MDC1 and ATR; however, after exposure (e.g. 12 h or 24 h after exposure), it is unknown whether further DNA damage accumulates or the accumulation is reduced and progresses toward repair. Experiments at 12-h and 24-h exposure times were not performed because the quantity and quality of total RNA after 12 h and 24 h varied. However, future studies involving cell transformation assay to estimate the carcinogenic potential of CSE are warranted.

In our study, the cytotoxicity of HTPs was similar to that of CCs, and the mechanism leading to carcinogenesis was suggested to be the repair pathways of DSBs involving MDC1 and ATR-CHK1. The carcinogenic substances in aerosols [N-nitrosonornicotine; 4-(methylnitrosamino)-1-(3-pyridyl)-1-butanone; 1,3-butadiene; benzene; formaldehyde; benzopyrene; o-toluidine; and 2-naphthylamine] are assumed to be responsible; however, we could not identify them, and further research is desirable.

## CONCLUSIONS

HTPs cause DNA DSBs and inhibit the MDC1 and ATR-CHK1 DNA repair pathways in primary HOKs. CCs have been reported to cause DNA damage and are related to carcinogenesis. Similarly, HTPs are genotoxic, can cause DSBs, and have toxicogenomic damage because they inhibit the MDC1 and ATR-CHK1 DNA repair pathways in the oral mucosa. Therefore, especially because the use of HTPs has increased among adolescents and young adults, it is important to realize that the use of HTPs from a young age also potentially increases the risk of DNA damage within the oral mucosa.

## Data Availability

The data supporting this research are available from the authors on reasonable request.
